# Association between B-cell activating factor and future depressive symptoms in hemodialysis patients

**DOI:** 10.47626/1516-4446-2025-4228

**Published:** 2025-12-18

**Authors:** Dong-Young Lee, Woojin Jang, Beom Kim, Jae-Hon Lee, Young Lee, Yu Ho Lee, Shin Young Ahn, Jin Sug Kim, Yang Gyun Kim, Hyeon Seok Hwang, Ju-Young Moon, Jae-Hong Ryoo, Kayla M. Teopiz, Rodrigo B. Mansur, Joshua D. Rosenblat, Roger S. McIntyre

**Affiliations:** 1 Department of Internal Medicine, Veterans Healthcare Service Medical Center, Seoul, Republic of Korea; 2 Department of Psychiatry, University of Toronto, Toronto, ON, Canada; 3 Department of Psychiatry, Schulich School of Medicine & Dentistry, Western University, London, ON, Canada; 4 Veterans Medical Research Institute, Veterans Healthcare Service Medical Center, Seoul, Republic of Korea; 5 Division of Nephrology, Department of Internal Medicine, CHA Bundang Medical Center, CHA University, Seongnam, Republic of Korea; 6 Department of Internal Medicine, College of Medicine, Korea University, Seoul, Republic of Korea; 7 Division of Nephrology, Department of Internal Medicine, Kyung Hee University, Seoul, Republic of Korea; 8 Departments of Occupational and Environmental Medicine, School of Medicine, Kyung Hee University, Seoul, Republic of Korea; 9 Mood Disorders Psychopharmacology Unit, Poul Hansen Family Centre for Depression, University of Health Network, Toronto, ON, Canada; 10 Department of Pharmacology and Toxicology, University of Toronto, Toronto, ON, Canada

**Keywords:** APRIL, BAFF, cytokines, depression, hemodialysis, TNF-α

## Abstract

**Objective:**

B-cell activating factor (BAFF) and a proliferation-inducing ligand (APRIL) are cytokines that play critical roles in the maturation, homeostasis, and differentiation of B-cells. Both cytokines have also been associated with mental disorders. The link between inflammation and depression is well-established. Patients undergoing hemodialysis who also experience depressive symptoms exhibit a state of immune dysfunction. We hypothesized that BAFF and APRIL levels would influence future depressive symptoms in hemodialysis patients.

**Methods:**

We enrolled 72 hemodialysis patients without baseline depressive symptoms. Depressive symptoms were assessed annually for 2 years using the Beck Depression Inventory-II. The participants were measured for plasma BAFF, APRIL, and tumor necrosis factor-α levels. To evaluate the impact of these levels on the development of depressive symptoms, we performed Cox regression and Kaplan-Meier analysis.

**Results:**

Depressive symptoms were observed in 31 (43.1%) patients. In both univariate and multivariate Cox regression analyses, a 1 SD increase in BAFF was significantly associated with an increased risk of future depressive symptoms, with hazard ratios of 1.44 (95%CI 1.03-2.00) and 1.70 (95%CI 1.04-2.78), respectively. Higher BAFF groups had a significantly greater incidence of depressive symptoms over 2 years (p = 0.048).

**Conclusion:**

Elevated plasma BAFF levels were significantly associated with the development of depressive symptoms in hemodialysis patients.

## Introduction

The number of dialysis patients has increased worldwide, doubling in South Korea in the last decade.^1^,^2^ People receiving dialysis often suffer from various comorbidities, such as cardiovascular diseases (CVD), chronic kidney disease (CKD)-mineral bone disorder, or anemia, many of which can increase mortality rates.^3^,^4^ However, through enormous efforts in early diagnosis and treatment of complications in end-stage renal disease, there has been a certain decrease in complications and mortality in dialysis patients.^2^ Depression, the most widespread psychiatric disorder among dialysis patients,^5^ remains an underexplored area of study, despite its significant impact on their well-being. The presence of comorbid depression in individuals undergoing dialysis has been associated with adverse clinical outcomes and may be mediated by factors such as malnutrition, reduced quality of life, and increased mortality risk.^6^-^8^ However, large-scale randomized controlled trials and meta-analyses have reported that the effects of antidepressants, such as selective serotonin reuptake inhibitors, on patients with end-stage renal disease who are undergoing dialysis and those with pre-dialysis CKD are neither significant nor consistent.^9^,^10^

Previous research has revealed a connection between inflammation and depression,^11^ with inflammation frequently emerging as a precursor to depressive episodes.^12^ Therefore, inflammation is one of the leading causes of depression.^12^ CKD is characterized by a persistent low-grade inflammatory state. As CKD progresses, the severity of inflammation increases.^13^ Inflammation in hemodialysis (HD) patients arises from a combination of uremic toxins, factors related to the dialysis process, and immune cell dysfunction.^14^,^15^ Taken together, it is hypothesized that antidepressants may be ineffective for depression in CKD patients because inflammation significantly contributes to the development of depression in this population.

The immune system of dialysis patients is dysregulated due to an imbalance of B-cells and T cells, in which some functions are activated while others are suppressed.^14^,^16^ B-cell activating factor (BAFF) and a proliferation-inducing ligand (APRIL) are members of the tumor necrosis factor (TNF) ligand superfamily.^16^,^17^ They play a crucial role in regulating the maturation, homeostasis, and differentiation of B-cells.^16^,^17^ Schizophrenia and bipolar disorder are also significantly associated with inflammation.^18^,^19^ Recent studies have found that BAFF and APRIL are significantly associated with schizophrenia and bipolar disorder.^20^,^21^ It is possible that, by modulating inflammation through B-cells, BAFF and APRIL could influence the development of schizophrenia and bipolar disorder. We hypothesize that BAFF and APRIL could also influence future depressive symptoms in chronic HD patients with immune dysfunction by regulating the immune system through B-cells.

## Methods

### Study population and design

**Figure 1:**
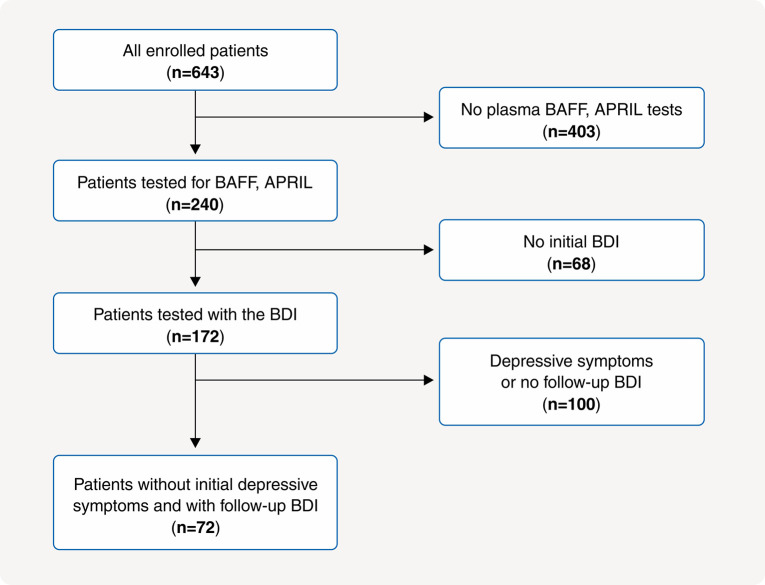
Flowchart of enrolled study participants. APRIL = a proliferation-inducing ligand; BAFF = B-cell activating factor; BDI = Beck Depression Inventory.

We conducted a prospective observational study with participants chosen from K-cohort, a multicenter HD cohort in South Korea in June 2016.^22^ Our main objective was to evaluate risk factors for comorbidity and mortality. The inclusion criteria were patients over the age of 18 years, having undergone more than 3 months of HD, and receiving HD treatment three times a week. The K-cohort’s exclusion criteria were patients with malignancies, hematologic disorders, or expected survival less than 6 months due to various medical conditions.^22^ We initially screened 643 patients and, after excluding those who did not undergo plasma TNF-α, BAFF, and APRIL testing, those who exhibited depressive symptoms according to the initial Korean-Beck Depression Inventory (K-BDI)-II scores, and those who failed to complete follow-up K-BDI assessments, 72 patients were included in the analysis (Figure 1). The institutional review board of each participating hospital approved the study, and informed consent was obtained from all involved patients. This study was registered with the World Health Organization clinical trials registry (KCT0003281).

### Study data and the primary outcome

We collected data on comorbidities, physical examinations, laboratory values, and HD-related information, applied depression questionnaires, and tested repository blood samples. Patient comorbidities included diabetes, hypertension, and CVD. The physical examinations consisted of body mass index and blood pressure. Most laboratory tests and HD-related data were collected as frequently as every 3 months. Annual questionnaires were applied over a 2-year period. The primary outcome of this study was the onset of depressive symptoms within 2 years, which was evaluated in relation to plasma levels of BAFF, APRIL, and TNF-α.

### Depressive symptoms

Professional medical staff administered the validated K-BDI-II to all participants annually,^23^ beginning at baseline and continuing for up to 2 years after enrollment. The 21 items of the K-BDI are scored from 0 to 3 (i.e., the highest possible score is 63). We considered total K-BDI scores > 16 to be indicative of depressive symptoms.^24^

### BAFF, APRIL, and TNF-α measurement

The biochemical parameters were measured using standard methods. We collected plasma samples in tubes treated with ethylenediaminetetraacetic acid on enrolment day. The samples were centrifuged at room temperature for 15 minutes at 1,000 *g* and were stored at -80 °C until analysis. We measured BAFF, APRIL, and TNF-α levels using an enzyme-linked immunosorbent assay with Magnetic Luminex Screening Assay multiplex kits (R&D Systems, Minneapolis, MN, USA), as described previously.^25^

### Statistical analysis

Continuous variables are presented as mean (SD) or median and interquartile range, depending on the results of the Shapiro-Wilk test for normality. Categorical variables are expressed as numbers (%). The subjects were classified into two groups according to the median BAFF and APRIL values. Differences in continuous variables between the groups were evaluated using an independent *t*-test for normally distributed variables and the Wilcoxon rank-sum test for non-normally distributed variables. Differences in categorical variables were assessed using the chi-square test. The study used the Kaplan-Meier method to determine cumulative depressive symptoms over 2 years according to BAFF, APRIL, and TNF-α levels, which were grouped as higher or lower according to the median value. We utilized Cox hazard ratio regression analysis to investigate the association between BAFF, APRIL, and TNF-α levels and the development of depressive symptoms within 2 years. To account for potential confounding factors, the Cox hazard ratio regression analyses were adjusted for age, sex, body mass index, diabetes, CVD, time on HD, spKt/V, and high-sensitive C-reactive protein. Variance inflation factors were assessed to check for multicollinearity among variables. The statistical analyses were performed in R 3.6.3.

## Results

The average patient age was 57.7 (SD, 12.9) years and 62.5% of the patients were men. Additionally, 34 (47.2%) patients had a history of CVD (Table 1). The mean and median baseline BDI scores were 8.8 (SD, 4.1) and 9.0 (6.0-12.0) points, respectively. The mean and median duration of HD were 60.6 (SD, 70.3) and 35.0 (13.0-76.0) months, respectively. The median BAFF, APRIL, and TNF-α levels were 800.8 (661.1-1002.9) pg/mL, 564.6 (410.9-830.2) pg/mL, and 7.74 (4.82-11.05) pg/mL, respectively. The body mass index, HD duration, BDI score, high-sensitive C-reactive protein, BAFF, and APRIL values did not meet the assumption of normality and were analyzed using non-parametric methods. Outliers were assessed using boxplots, and values falling outside 1.5 times the interquartile range from the first and third quartiles were examined. All outliers were judged to be clinically plausible and were retained in the analysis.

No significant differences were found for any variable between the lower and higher BAFF groups, which were classified according to a median BAFF value of 800.75 pg/mL. However, the higher BAFF group had significantly higher APRIL levels (p < 0.001) (Table 1). Between the lower and higher APRIL groups, classified according to a median APRIL value of 564.6 pg/mL, there were no significant differences in any variables, except significantly higher BAFF levels higher APRIL group (p < 0.001) (Table 2). Both BAFF and APRIL were significantly correlated (r = 0.356, p = 0.002).

**Figure 2:**
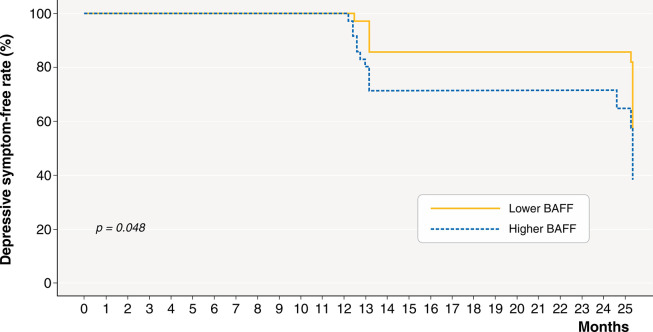
Depressive symptom-free rate according to plasma BAFF level. BAFF = B-cell activating factor.

During 2 years of follow-up, depressive symptoms were found in 31 (43.1%) patients. The cumulative depressive symptoms were significantly greater in the higher BAFF group (Figure 2).

According to the results of the univariate Cox regression analysis, there was a significant association between BAFF level and the occurrence of depressive symptoms within 2 years. The hazard ratio was 1.437 per 1 SD increment, with a 95%CI of 1.034-1.996 (Table 3). Even after adjusting for multiple variables, this association remained significant, with a hazard ratio of 1.699 per 1 SD increment and a 95%CI of 1.037-2.784. However, there was no significant association between APRIL or TNF-α and depressive symptoms within 2 years. All variables demonstrated variance inflation factors values < 5, indicating no significant multicollinearity.

## Discussion

According to our results, HD patients without depressive symptoms experienced a 43.1% incidence of depressive symptoms within 2 years. Plasma BAFF levels were significantly associated with incident depressive symptoms within 2 years in univariate and multivariate Cox regression analyses. Moreover, the cumulative incidence of depressive symptoms was significantly greater in the higher BAFF group in the Kaplan-Meier survival analysis. Although plasma TNF-α is a well-known proinflammatory cytokine associated with depression in the general population, it was not related to future depressive symptoms in our sample.

Our methodology has several strengths. First, we focused on predicting depressive symptoms over a 2-year period based on plasma BAFF, APRIL, and TNF-α levels. Research on depression and biomarkers, such as BAFF and APRIL, is currently limited. Moreover, few studies have explored the potential to predict future depression based on these specific biomarkers. Secondly, as there are few studies that have evaluated long-term depressive symptoms in dialysis patients,^26^ our longer observation period of 2 years represents a key strength. Further, during this 2-year prospective study, a relatively large number of patients were enrolled and completed the study. Moreover, few studies have reported the pathophysiological mechanisms of depressive symptoms in HD patients.

It has been postulated that the etiology of depression in HD patients can be divided into behavioral and biological aspects.^27^ Regarding behavior, patients undergoing HD frequently visit hospitals, carry a high pill burden, face dietary restrictions, and feel pressured to self-manage their blood pressure, blood sugar, and body weight, all of which may lead to symptoms of depression.^27^ Visiting HD units three times a week could be burdensome for patients and could increase their mortality risk.^28^ Additionally, long HD duration has been identified as a risk factor for depression.^29^ It has been reported that functional impairment and end-stage renal disease symptoms can also contribute to the development of depression.^30^ Biologically, dialysis-independent or dependent CKD is a somewhat inflammatory state, which is associated with depression. Furthermore, diabetes and hypertension, which are not only the major causes of CKD but are frequently comorbid in CKD, also involve an inflammatory state.^27^ Research has shown that individuals suffering from depression may be at a higher risk of developing CVD and experiencing a poorer prognosis for existing cardiovascular conditions.^31^ Taken together, the evidence indicates that depression and CKD (including end-stage renal disease) have a bidirectional relationship.^27^

Some studies have reported that HD patients with depression have increased levels of pro-inflammatory cytokines, such as interleukin (IL)-1, IL-6, and TNF-α, along with elevated levels of acute phase proteins, such as ferritin.^32^,^33^ Additionally, they show decreased levels of IL-10, an anti-inflammatory cytokine, and albumin, which is a negative acute phase protein. The mechanism by which IL-6 contributes to depression is believed to involve the activation of the hypothalamic-pituitary-adrenal axis, leading to increased cortisol and corticotropin-releasing hormone, as well as the upregulation of indoleamine 2,3-dioxygenase and neuroinflammation.^12^ This neuroinflammation disrupts neural circuits and activates astrocytes and microglia. TNF-α contributes to depression by modifying glutamate signaling, decreasing neuroplasticity (e.g., brain-derived neurotrophic factor), and increasing anhedonia and fatigue.^12^ It has been hypothesized that peripheral inflammation could induce depression by transmitting signals to the brain through various pathways.^34^ Bobot et al.^35^ reported that uremic toxins like indoxyl sulfate could activate the aryl hydrocarbon receptor by disrupting the blood-brain barrier, while Hernandez et al.^36^ demonstrated that blood-brain barrier and gut-blood barrier disruptions exist in CKD patients. Taken together, it can be extrapolated that end-stage renal disease patients are more vulnerable to depression due to disruptions in the blood-brain and gut-brain barriers, through which peripheral inflammatory cytokines could penetrate the brain, in addition to existing pathways.

Inflammation in HD patients is characterized by an unbalanced immune system, which results in hyperactivation (such as increased cytokine production) and immunosuppression (such as decreased immune cell function). This imbalance leads to high rates of infection and poor vaccine response (35807042, 30876622).^37^,^38^ In patients undergoing HD, both the number of B-cells and the production of IgG and IgM are reduced. In addition, there is an increase in the number of memory B-cells and elevated levels of IL-6 and TNF-α, along with increased autoantibody production.^37^,^39^,^40^ These changes may result in neuroinflammation, dysregulation of neurotransmitters, such as indoleamine 2,3-dioxygenase and serotonin, autoantibody production, and an inability to suppress inflammation.^32^,^37^ BAFF significantly affects the development and function of B-cells.^16^,^17^ BAFF is believed to contribute to depression in HD patients due to its potential to cause neuroinflammation, affect the production of autoantibodies and immune complexes, and disrupt the hypothalamic-pituitary-adrenal axis, in addition to its direct effects on the central nervous system.^41^-^43^

In addition to dialysis, several chronic medical conditions, such as diabetes and CVD, are significantly associated with depression.^44^,^45^ The link between depression and these conditions may be influenced by several factors, including neuroinflammation and cytokine dysregulation, hypothalamic-pituitary-adrenal axis dysregulation, oxidative stress and mitochondrial dysfunction, and vascular and neurostructural changes, in addition to psychosocial and behavioral factors.^46^-^48^ Thus, the inflammation caused by these medical conditions may initiate the disease process.

In the National Ambulatory Medical Care Survey, which investigated primary care in the United States, the overall rate of depression screening was just 4.2%, even though screening for depression was recommended as a part of routine primary care.^49^ The depression screening rate among older adults was half that among middle aged adults.^49^ Of the 240 patients who were tested for plasma BAFF and APRIL, 68 did not complete the BDI. According to the Dialysis Outcomes and Practice Patterns Study, the prevalence of depressive symptoms among dialysis patients is as high as 40%,^5^ and depressive symptoms tend to be underdiagnosed and undertreated in HD patients, which reflects contemporary trends among HD patients worldwide.^50^ In line with this, HD patients aged 65 years or more are less likely to be screened for depressive symptoms, so the reported prevalence of depression in HD patients might be underestimated. Because it is much easier to test plasma BAFF than administer depression questionnaires, testing plasma BAFF may help diagnose and manage depressive symptoms in HD patients.

Some study limitations could affect the interpretation of our data. First, we did not investigate patients diagnosed with depression and who were prescribed medication, as the depressive symptoms of some participants may have improved after taking antidepressants. Second, we did not conduct a flow cytometry test on the B-cell subpopulation. Considering the function of BAFF and APRIL on B-cells, flow cytometry may have helped explain the difference in B-cell subpopulations according to the BAFF and APRIL levels. Third, we did not measure BAFF or APRIL levels at the time of the BDI questionnaire in years 1 or 2 of the study. Fourth, although the number of enrolled patients was relatively large, a larger population than 72 is needed to prove our hypothesis.

In conclusion, we found that a high plasma BAFF level was significantly associated with the occurrence of depressive symptoms within 2 years in HD patients. Moreover, the higher BAFF group had a greater cumulative occurrence of depressive symptoms within 2 years. These results suggest that plasma BAFF levels might be useful as a predictor of depressive symptoms.

Disclosure

RSM has received research grant support from the Canadian Institutes of Health Research (CIHR)/the Global Alliance for Chronic Diseases (GACD)/the National Natural Science Foundation of China (NSFC) and the Milken Institute, as well as speaker/consultation fees from Lundbeck, Janssen, Alkermes, Neumora Therapeutics, Boehringer Ingelheim, Sage, Biogen, Mitsubishi Tanabe, Purdue, Pfizer, Otsuka, Takeda, Neurocrine, Sunovion, Bausch Health, Axsome, Novo Nordisk, Kris, Sanofi, Eisai, Intra-Cellular, NewBridge Pharmaceuticals,Viatris, Abbvie, Atai Life Sciences. RSM is a CEO of Braxia Scientific Corp. KMT has received fees from Braxia Scientific Corp. The other authors report no conflicts of interest.

## Figures and Tables

**Table 1:** The baseline characteristics of all patients according to BAFF level

	Total (n = 72)	Lower BAFF group < 800.75 (pg/mL)	Higher BAFF group ≥ 800.75 (pg/mL)		
Variables	Mean ± SD or median (IQR)	Mean ± SD or median (IQR)	Mean ± SD or median (IQR)	Statistics	p-value
Age (years)	57.7 ± 12.9	57.9 ± 13.6	60.4 ± 12.1	t_(70)_ = -1.827	0.072
BMI (kg/m^2^)	21.9 (20.2-25.1)	22.6 (20.3-25.0)	21.4 (20.2-25.1)	W = 682	0.708
HD duration (months)	35.0 (13.0-76.0)	33.5 (13.0-81.0)	39.0 (13.0-75.0)	W = 373.5	0.682
spKt/V	1.58 ± 0.31	1.60 ± 0.29	1.56 ± 0.32	t_(70)_ = 0.599	0.551
BDI score	9.0 (6.0-12.0)	9.0 (5.0-12.5)	9.5 (6.0-11.5)	W = 645	0.977
hsCRP (mg/L)	0.80 (0.24-2.46)	0.66 (0.20-2.46)	0.96 (0.39-2.46)	W = 571	0.389
BAFF (pg/mL)	800.8 (661.1-1002.9)	661.1 (538.1-725.1)	1002.9 (891.3-1205.4)	W = 0	< 0.001
APRIL (pg/mL)	564.6 (410.9-830.2)	443.2 (325.3-622.4)	685.7 (543.2-1024.4)	W = 307	< 0.001
TNF-α (pg/mL)	8.12 ± 4.42	7.76 ± 4.31	8.48 ± 4.56	t(70) = -0.683	0.497
Men	45 (62.5)	25 (69.4)	20 (55.6)	χ^2^_(1)_ = 0.948	0.33
Diabetes	44 (61.1)	18 (50.0)	26 (72.2)	χ^2^_(1)_ = 2.864	0.091
CVD	34 (47.2)	18 (50.0)	16 (44.4)	χ^2^_(1)_ = 0.056	0.813

Categorical variables were presented as n (%). Continuous variables were presented as mean ± SD when normally distributed and as median (interquartile range [IQR]) when non- normally distributed. Group comparisons were performed using an independent *t*-test for normally distributed continuous variables, the Wilcoxon rank-sum test for non-normally distributed continuous variables, and the chi-square test for categorical variables. Corresponding test statistics are presented as t(degrees of freedom [df]), W, or χ^2^(df) values as appropriate. APRIL = a proliferation-inducing ligand; BAFF = B-cell activating factor; BDI = Beck Depression Inventory; BMI = body mass index; CVD = cardiovascular diseases; HD = hemodialysis; hsCRP = high sensitivity C-reactive protein; TNF-α = tumor necrosis factor-α.

**Table 2:** The baseline characteristics of all patients according to APRIL level

	Lower APRIL Group <564.6 (pg/mL)	Higher APRIL Group ≥564.6 (pg/mL)		
Variables	Mean ± SD or Median (IQR)	Mean ± SD or Median (IQR)	Statistics	p-value
Age (years)	57.6 ± 13.7	57.8 ± 12.2	t_(70)_ = -0.045	0.964
BMI (kg/m^2^)	22.0 (20.0-25.2)	21.8 (20.6-25.0)	W = 614	0.708
HD duration (months)	37.0 (15.0-110.0)	25.5 (12.0-61.0)	W = 418	0.214
spKt/V	1.60 ± 0.26	1.56 ± 0.35	t_(70)_ = 0.645	0.521
BDI score	8.5 (5.0-12.0)	10.0 (6.0-12.0)	W = 620	0.756
hsCRP (mg/L)	0.73 (0.21-2.88)	0.88 (0.39-1.73)	W = 655.5	0.937
BAFF (pg/mL)	725.1 (585.4-861.5)	891.3 (721.1-1143.0)	W = 382	0.002
APRIL (pg/mL)	410.9 (325.3-513.9)	830.2 (662.1-1090.2)	W = 0	< 0.001
TNF-α (pg/mL)	7.74 ± 4.29	8.50 ± 4.58	t_(70)_ = -0.724	0.472
Male	23 (63.9)	22 (61.1)	χ^2^_(1)_ = 0	1
Diabetes	20 (55.6)	24 (66.7)	χ^2^_(1)_ = 0.526	0.468
Cardiovascular disease	14 (38.9)	20 (55.6)	χ^2^_(1)_ = 1.393	0.238

Categorical variables are presented as n (%). Continuous variables are presented as mean ± SD when normally distributed and as median (interquartile range [IQR]) when not. Group comparisons were performed using an independent *t*-test for normally distributed continuous variables, the Wilcoxon rank-sum test for non-normally distributed continuous variables, and the chi-square test for categorical variables. Corresponding test statistics are presented as t(degrees of freedom [df]), W, or χ^2^(df) values as appropriate. APRIL = a proliferation-inducing ligand; BAFF = B-cell activating factor; BDI = Beck Depression Inventory; BMI = body mass index; HD = hemodialysis; hsCRP = high sensitivity C-reactive protein; TNF-α = tumor necrosis factor-α.

**Table 3:** HR of plasma BAFF, APRIL, and TNF- α for depressive symptoms

Variables	Crude HR (95%CI)	p-value	Adjusted HR (95%CI)	p-value
BAFF (pg/mL)	1.44 (1.03-2.00)	0.030	1.70 (1.04-2.78)	0.035
APRIL (pg/mL)	0.84 (0.57-1.25)	0.390	0.96 (0.64-1.44)	0.836
TNF-α (pg/mL)	0.90 (0.62-1.32)	0.604	0.84 (0.53-1.36)	0.493

Adjusted for age, sex, body mass index, diabetes, cardiovascular diseases, hemodialysis, spKt/V, and high-sensitive C-reactive protein. APRIL = a proliferation-inducing ligand; BAFF = B-cell activating factor; HR = hazard ratio; TNF-α = tumor necrosis factor-α.

## Data Availability

The data that support this study are available from the authors upon request.
